# Supplementary material for: Diversification rate vs. diversification density: Decoupled consequences of plant height for diversification of Alooideae in time and space
Source: PLoS One. 2020 May 26;15(5):e0233597. doi: 10.1371/journal.pone.0233597 (PMC7250425; doi:10.1371/journal.pone.0233597)
Supplement: S1 Appendix — (DOC) [file pone.0233597.s001.doc]

**APPENDICES FOR:**

**DIVERSIFICATION RATE VS. DIVERSIFICATION DENSITY: DECOUPLED CONSEQUENCES OF PLANT HEIGHT FOR DIVERSIFICATION OF ALOOIDEAE IN TIME AND SPACE**

**APPENDIX S1: PHYLOGENETIC INFERENCE**

**
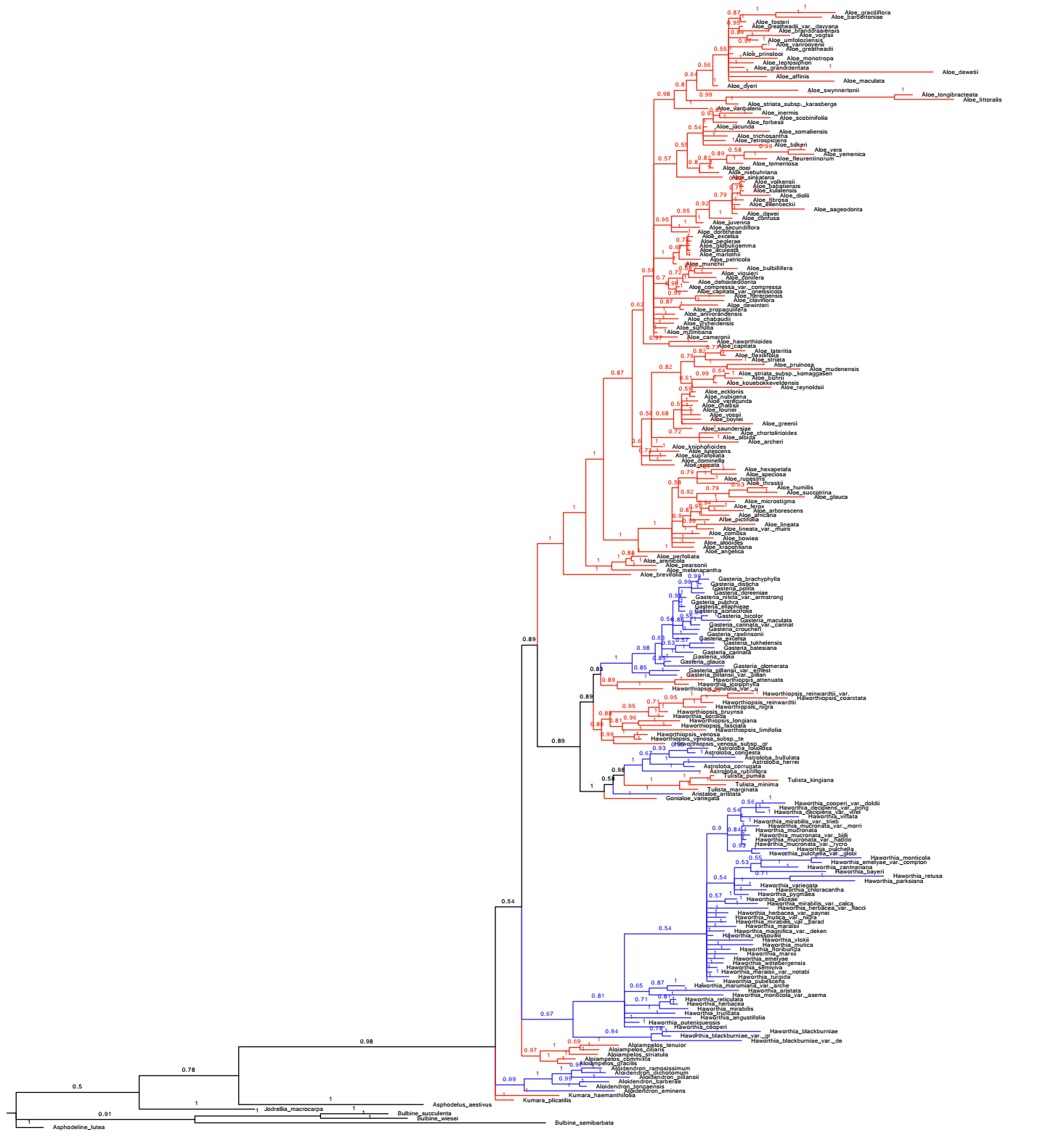
**

**Figure S1. Phylogenetic hypothesis for Alooideae.** The figure shows the majority-rule consensus phylogram from the phylogenetic posterior, with posterior probabilities shown on each branch. Genera have been colored in red and blue alternatively so that they can be easily distinguished.

**Table S1. Accession numbers for sequences used in this study.**

Accessions number starting with ‘GI’ are NCBI GI numbers (<https://www.ncbi.nlm.nih.gov/genbank/sequenceids/>). Accession numbers starting with other letters are NCBI accession numbers (<https://www.ncbi.nlm.nih.gov/Sequin/acc.html>). Cells with ‘NA’ show missing sequences.

| ***Species*** | **matK** | **psbA** | **rbcL** | **trnL** | **trnLF** | **ITS1** | **rsp16** |
| --- | --- | --- | --- | --- | --- | --- | --- |
| ***Aloe_aageodonta*** | KJ557716.1 | NA | GI1007357615 | NA | NA | NA | KJ557472 |
| ***Aloe_aculeata*** | KJ557717.1 | NA | GI555630831 | NA | KF013436.1 | NA | KJ557473 |
| ***Aloe_affinis*** | KJ557718.1 | NA | NA | KJ557628.1 | NA | NA | KJ557474 |
| ***Aloe_africana*** | JX518056.1 | GI345285497 | GI409975579 | NA | KF013437.1 | GI345285544 | NA |
| ***Aloe_albida*** | JX517401.1 | NA | GI409975581 | NA | NA | GI388268860 | NA |
| ***Aloe_alooides*** | JX518239.1 | NA | GI409975583 | NA | JX630286.1 | GI388268819 | NA |
| ***Aloe_angelica*** | JQ024109.1 | NA | NA | NA | JX630327.1 | GI388268804 | NA |
| ***Aloe_anivoranoensis*** | JX517497.1 | NA | GI409975585 | NA | JX630285.1 | GI388268865 | NA |
| ***Aloe_arborescens*** | GI37361782 | NA | GI37361668 | NA | KP072697.1 | GI388268820 | KJ557477 |
| ***Aloe_archeri*** | KU748258.1 | NA | GI1007357585 | NA | NA | NA | KJ557478 |
| ***Aloe_arenicola*** | KJ557721.1 | GI371436903 | GI371437927 | NA | JX630328.1 | GI388268762 | KJ557479 |
| ***Aloe_babatiensis*** | KJ557723.1 | NA | GI555630837 | NA | KF013441.1 | NA | KJ557481 |
| ***Aloe_bakeri*** | NA | NA | GI1332424 | AJ290254.1 | AJ290288.1 | NA | NA |
| ***Aloe_barbertoniae*** | KJ557724.1 | NA | NA | KJ557632.1 | NA | NA | KJ557482 |
| ***Aloe_bowiea*** | KU937284.1 | GI345285494 | GI371437937 | NA | HQ646842.1 | GI345285541 | NA |
| ***Aloe_boylei*** | GI33635888 | NA | GI33636026 | NA | NA | NA | KJ557484 |
| ***Aloe_branddraaiensis*** | KJ557726.1 | NA | NA | KJ557633.1 | NA | NA | KJ557485 |
| ***Aloe_brevifolia*** | JQ024117.1 | NA | GI371437939 | NA | JX630273.1 | GI388268808 | KJ557487 |
| ***Aloe_buhrii*** | JQ024118.1 | NA | GI371437941 | NA | JX630257.1 | GI388268757 | NA |
| ***Aloe_bulbillifera*** | GI33635870 | NA | GI33636014 | NA | KF013440.1 | NA | KJ557489 |
| ***Aloe_cameronii*** | KJ557732.1 | NA | NA | KJ557634.1 | NA | NA | KJ557491 |
| ***Aloe_capitata*** | AY323720.1 | NA | GI37361664 | NA | NA | GI37361705 | NA |
| ***Aloe_chabaudii*** | KJ557735.1 | GI388268410 | GI555630853 | NA | JX630303.1 | GI388268793 | KJ557495 |
| ***Aloe_challisii*** | JX517888.1 | NA | GI409975595 | NA | JX630283.1 | GI388268849 | NA |
| ***Aloe_chortolirioides*** | JX518123.1 | NA | GI409975597 | NA | JX630287.1 | GI388268868 | NA |
| ***Aloe_claviflora*** | KJ557738.1 | NA | NA | KJ557635.1 | NA | NA | KJ557498 |
| ***Aloe_comosa*** | JQ024124.1 | GI388268418 | GI371437949 | NA | NA | GI388268822 | KJ557499 |
| ***Aloe_compressa*** | GI37361780 | NA | GI37361666 | NA | NA | GI37361706 | NA |
| ***Aloe_confusa*** | KU748288.1 | NA | GI1005352136 | NA | KF013454.1 | NA | KJ557501 |
| ***Aloe_conifera*** | GI33635866 | NA | GI33636010 | NA | KF013449.1 | GI37361707 | KJ557502 |
| ***Aloe_dawei*** | KC893701.1 | NA | GI1005352138 | NA | NA | NA | KJ557504 |
| ***Aloe_deltoideodonta*** | GI33635868 | NA | GI33636012 | NA | KF013459.1 | NA | KJ557506 |
| ***Aloe_dewetii*** | KJ557746.1 | NA | NA | KJ557637.1 | NA | NA | KJ557508 |
| ***Aloe_dewinteri*** | JQ024125.1 | GI388268420 | GI371437953 | NA | JX630249.1 | GI388268797 | NA |
| ***Aloe_diolii*** | KJ557747.1 | NA | GI1005352144 | NA | KF013456.1 | NA | KJ557509 |
| ***Aloe_doei*** | AY323724.1 | NA | GI37361672 | NA | NA | GI37361710 | NA |
| ***Aloe_dominella*** | KJ557749.1 | NA | GI409975601 | NA | NA | NA | KJ557511 |
| ***Aloe_dorotheae*** | KU748290.1 | NA | GI1005352062 | NA | NA | NA | KJ557512 |
| ***Aloe_dyeri*** | JQ024129.1 | NA | GI371437961 | NA | KF013460.1 | NA | NA |
| ***Aloe_ecklonis*** | JX517611.1 | GI388268426 | GI409975603 | NA | JX630277.1 | GI388268801 | KJ557513 |
| ***Aloe_ellenbeckii*** | KJ557752.1 | NA | GI555630861 | KJ557638.1 | KF013457.1 | NA | KJ557514 |
| ***Aloe_excelsa*** | KC893703.1 | GI388268430 | GI326393933 | NA | JX630302.1 | GI388268795 | KJ557516 |
| ***Aloe_ferox*** | KC893704.1 | NA | GI371436982 | NA | JX630259.1 | GI388268821 | KJ557518 |
| ***Aloe_fibrosa*** | KJ557755.1 | NA | GI555630879 | NA | KF013468.1 | NA | KJ557519 |
| ***Aloe_fleurentinorum*** | KJ557756.1 | NA | NA | NA | KF013469.1 | NA | KJ557520 |
| ***Aloe_flexilifolia*** | KJ557757.1 | NA | GI1005352096 | NA | NA | NA | KJ557521 |
| ***Aloe_forbesii*** | GI33635878 | NA | GI33636020 | KJ557639.1 | NA | GI37361716 | KJ557522 |
| ***Aloe_fosteri*** | JQ024131.1 | NA | GI371437965 | KJ557640.1 | NA | NA | KJ557523 |
| ***Aloe_fouriei*** | JX517684.1 | NA | GI409975609 | NA | JX630281.1 | GI388268852 | NA |
| ***Aloe_glauca*** | GI33635892 | NA | GI33636030 | NA | JX630264.1 | GI388268807 | KJ557526 |
| ***Aloe_globuligemma*** | KJ557763.1 | NA | GI371437971 | NA | KF013471.1 | NA | KJ557527 |
| ***Aloe_graciliflora*** | KJ557764.1 | NA | NA | KJ557641.1 | NA | NA | KJ557528 |
| ***Aloe_grandidentata*** | KJ557765.1 | NA | GI555630883 | KJ557642.1 | KF013470.1 | NA | KJ557529 |
| ***Aloe_greatheadii*** | KJ557766.1 | GI388268440 | GI563425622 | KJ557643.1 | NA | NA | KJ557530 |
| ***Aloe_greenii*** | KJ557767.1 | NA | NA | KJ557644.1 | NA | NA | KJ557531 |
| ***Aloe_haworthioides*** | JQ024139.1 | NA | GI371437979 | NA | JX630186.1 | GI388268851 | KJ557532 |
| ***Aloe_hereroensis*** | JQ024140.1 | GI388268444 | GI371437981 | NA | JX630251.1 | GI388268799 | KJ557533 |
| ***Aloe_hexapetala*** | JQ024141.1 | NA | GI371437983 | NA | NA | GI388268812 | NA |
| ***Aloe_humilis*** | AY323719.1 | NA | GI37361662 | NA | NA | GI37361703 | KJ557535 |
| ***Aloe_inermis*** | GI33635874 | NA | GI33635980 | KJ557645.1 | NA | GI37361714 | KJ557536 |
| ***Aloe_jucunda*** | GI37361774 | NA | GI37361660 | KJ557646.1 | NA | GI37361702 | KJ557537 |
| ***Aloe_juvenna*** | GI37361772 | NA | GI37361658 | NA | KF013478.1 | GI37361701 | KJ557538 |
| ***Aloe_kniphofioides*** | JX517649.1 | NA | GI595745010 | NA | KC985128.1 | NA | NA |
| ***Aloe_kouebokkeveldensis*** | JQ024144.1 | NA | GI371437989 | NA | JX630245.1 | GI388268758 | NA |
| ***Aloe_krapohliana*** | KJ557777.1 | NA | NA | NA | NA | NA | KJ557544 |
| ***Aloe_kulalensis*** | KJ557778.1 | NA | GI555630899 | NA | KF013479.1 | NA | KJ557545 |
| ***Aloe_lateritia*** | KJ557779.1 | NA | NA | KJ557647.1 | NA | NA | KJ557546 |
| ***Aloe_leptosiphon*** | KJ557781.1 | NA | NA | KJ557648.1 | NA | NA | KJ557548 |
| ***Aloe_lineata*** | GI33635894 | NA | GI371437991 | NA | HQ646846.1 | GI388268814 | NA |
| ***Aloe_littoralis*** | KJ557783.1 | NA | GI563425620 | NA | NA | NA | KJ557550 |
| ***Aloe_longibracteata*** | KJ557784.1 | NA | NA | KJ557649.1 | NA | NA | KJ557551 |
| ***Aloe_lutescens*** | NA | GI388268452 | NA | NA | JX630330.1 | GI388268842 | NA |
| ***Aloe_maculata*** | KP149511.1 | NA | GI379133813 | KJ557651.1 | KP072700.1 | NA | KJ557553 |
| ***Aloe_marlothii*** | KC893708.1 | NA | GI326393935 | NA | KF013485.1 | NA | KJ557554 |
| ***Aloe_melanacantha*** | JQ024150.1 | GI388268454 | GI371437999 | NA | JX630274.1 | GI388268761 | KJ557557 |
| ***Aloe_microstigma*** | KJ557789.1 | NA | GI371438003 | NA | JX630253.1 | GI388268817 | KJ557558 |
| ***Aloe_monotropa*** | KJ557791.1 | NA | NA | KJ557652.1 | NA | NA | KJ557560 |
| ***Aloe_mudenensis*** | KJ557793.1 | NA | GI555630915 | KJ557653.1 | KF013488.1 | NA | KJ557562 |
| ***Aloe_munchii*** | JX517965.1 | GI388268458 | GI409975621 | NA | JX630282.1 | GI388268796 | NA |
| ***Aloe_mzimbana*** | KJ557794.1 | NA | GI555630919 | NA | KF013490.1 | NA | KJ557563 |
| ***Aloe_niebuhriana*** | GI37361788 | NA | GI37361674 | NA | KF013491.1 | GI37361711 | M35112 |
| ***Aloe_nubigena*** | JX518145.1 | NA | GI409975623 | NA | JX630239.1 | GI388268850 | NA |
| ***Aloe_pearsonii*** | KC893709.1 | GI371436908 | GI371438005 | NA | JX630325.1 | GI388268763 | KJ557566 |
| ***Aloe_peglerae*** | JX517749.1 | NA | GI409975625 | NA | NA | NA | KJ557567 |
| ***Aloe_perfoliata*** | JQ024156.1 | GI388268462 | GI371438009 | NA | JX630322.1 | GI388268809 | NA |
| ***Aloe_petricola*** | JQ024157.1 | GI388268464 | GI371438011 | NA | JX630326.1 | GI388268794 | KJ557571 |
| ***Aloe_pictifolia*** | KC893710.1 | NA | GI371438013 | KJ557654.1 | JX630248.1 | GI388268818 | KJ557572 |
| ***Aloe_prinslooi*** | KJ557802.1 | NA | NA | KJ557655.1 | NA | NA | KJ557574 |
| ***Aloe_propagulifera*** | JX517367.1 | NA | GI409975631 | NA | JX630284.1 | GI388268853 | NA |
| ***Aloe_pruinosa*** | KJ557803.1 | NA | NA | KJ557656.1 | NA | NA | KJ557575 |
| ***Aloe_retrospiciens*** | KJ557805.1 | NA | GI555630925 | NA | KF013495.1 | NA | KJ557577 |
| ***Aloe_reynoldsii*** | JQ024160.1 | GI371436909 | GI371438017 | KJ557657.1 | JX630246.1 | GI388268759 | KJ557578 |
| ***Aloe_rupestris*** | JX517584.1 | GI388268472 | NA | NA | JX630280.1 | GI388268811 | NA |
| ***Aloe_saundersiae*** | JX517720.1 | GI388268474 | GI409975635 | NA | JX630275.1 | GI388268839 | NA |
| ***Aloe_scobinifolia*** | GI33635876 | NA | GI33636018 | KJ557659.1 | KF013502.1 | GI37361715 | NA |
| ***Aloe_secundiflora*** | KU748257.1 | NA | GI1007357583 | NA | NA | NA | KJ557581 |
| ***Aloe_sinkatana*** | GI33635872 | NA | GI33636016 | KJ557661.1 | KF013503.1 | GI37361717 | KJ557583 |
| ***Aloe_somaliensis*** | KJ557810.1 | NA | NA | NA | KF013501.1 | NA | KJ557584 |
| ***Aloe_speciosa*** | KJ557811.1 | GI345285496 | GI555630931 | NA | KF013498.1 | GI345285543 | KJ557585 |
| ***Aloe_spicata*** | KC893712.1 | NA | GI326393937 | NA | JX630301.1 | GI388268784 | KJ557586 |
| ***Aloe_striata*** | GI33635884 | GI371436910 | GI33636024 | KJ557662.1 | JX630256.1 | GI388268754 | KJ557587 |
| ***Aloe_succotrina*** | KC893714.1 | GI371436913 | GI371438031 | NA | JX630266.1 | GI388268760 | KJ557588 |
| ***Aloe_suffulta*** | KJ557814.1 | NA | NA | KJ557663.1 | NA | NA | KJ557589 |
| ***Aloe_suprafoliata*** | GI37361768 | NA | GI37361654 | NA | KF013504.1 | GI37361704 | KJ557590 |
| ***Aloe_swynnertonii*** | KJ557815.1 | NA | NA | KJ557664.1 | NA | NA | KJ557591 |
| ***Aloe_thraskii*** | KJ557816.1 | GI388268482 | GI371438037 | NA | JX630261.1 | GI388268813 | KJ557592 |
| ***Aloe_tomentosa*** | KC893716.1 | NA | GI555630939 | NA | KF013506.1 | NA | KJ557593 |
| ***Aloe_trichosantha*** | KJ557817.1 | NA | GI555630943 | NA | KF013508.1 | NA | KJ557594 |
| ***Aloe_umfoloziensis*** | KJ557819.1 | NA | NA | KJ557665.1 | NA | NA | KJ557596 |
| ***Aloe_vanbalenii*** | KJ557820.1 | NA | GI555630947 | KJ557666.1 | KF013510.1 | NA | KJ557597 |
| ***Aloe_vanrooyenii*** | KJ557821.1 | NA | NA | KJ557667.1 | NA | NA | KJ557598 |
| ***Aloe_vera*** | GI372485171 | NA | GI33636022 | AJ290255.1 | KP072706.1 | NA | KJ557601 |
| ***Aloe_verecunda*** | GI33635890 | NA | GI33636028 | NA | JX630271.1 | GI388268840 | NA |
| ***Aloe_viguieri*** | GI33635864 | NA | GI33636008 | NA | KF013512.1 | NA | KJ557602 |
| ***Aloe_vogtsii*** | KJ557823.1 | NA | GI555630953 | KJ557668.1 | KF013514.1 | NA | KJ557603 |
| ***Aloe_volkensii*** | KJ557824.1 | NA | GI1005352082 | NA | NA | NA | KJ557604 |
| ***Aloe_vossii*** | JX518216.1 | GI388268486 | GI409975641 | NA | JX630276.1 | GI388268841 | NA |
| ***Aloe_vryheidensis*** | JX517863.1 | GI388268488 | GI409975643 | NA | JX630279.1 | GI388268802 | NA |
| ***Aloe_yemenica*** | KJ557826.1 | NA | NA | NA | KF013516.1 | NA | KJ557606 |
| ***Aloiampelos_ciliaris*** | GI33635858 | GI371436906 | GI33635978 | KJ557669.1 | JX630267.1 | GI388268786 | KJ557608 |
| ***Aloiampelos_commixta*** | JQ024122.1 | GI388268416 | GI371437947 | KJ557670.1 | JX630258.1 | GI388268823 | KJ557609 |
| ***Aloiampelos_gracilis*** | JQ024137.1 | GI388268438 | GI371437973 | NA | JX630255.1 | GI388268824 | NA |
| ***Aloiampelos_striatula*** | NA | NA | NA | NA | JX630187.1 | NA | KJ557610 |
| ***Aloiampelos_tenuior*** | JQ024169.1 | GI388268480 | GI371438033 | NA | JX630254.1 | GI388268825 | NA |
| ***Aloidendron_barberae*** | JQ024115.1 | NA | GI371437931 | KJ557671.1 | JX630188.1 | GI388268756 | KJ557611 |
| ***Aloidendron_dichotomum*** | JQ024126.1 | NA | GI371437955 | NA | JX630323.1 | JQ025368.1 | KJ557612 |
| ***Aloidendron_eminens*** | JX518151.1 | NA | GI409975605 | NA | JX630329.1 | GI388268863 | NA |
| ***Aloidendron_pillansii*** | GI33635838 | NA | GI33635988 | NA | JX630252.1 | GI37361687 | KJ557613 |
| ***Aloidendron_ramosissimum*** | AJ511370.1 | NA | GI33635990 | NA | JX630324.1 | GI37361688 | KJ557614 |
| ***Aloidendron_tongaensis*** | KX146280.1 | NA | GI1066800736 | NA | NA | NA | NA |
| ***Aristaloe_aristata*** | AY323713.1 | GI388268406 | NA | KJ557630.1 | JX630272.1 | GI388268806 | KJ557480 |
| ***Asphodeline_lutea*** | GI33635932 | NA | NA | AJ290256.1 | NA | NA | NA |
| ***Asphodelus_aestivus*** | GI33635930 | NA | GI313756477 | AJ290257.1 | NA | NA | NA |
| ***Astroloba_bullulata*** | JQ024172.1_ | NA | GI371438041 | NA | HQ646838.1 | GI345285538 | NA |
| ***Astroloba_corrugata*** | AJ511410.1_ | NA | GI33635964 | NA | JX630201.1 | GI388268844 | NA |
| ***Astroloba_foliolosa*** | KJ557834.1_ | NA | GI371438047 | NA | KF013534.1 | GI388268845 | KJ557615 |
| ***Astroloba_herrei*** | JQ024176.1_ | NA | GI371438049 | NA | JX630206.1 | GI388268843 | NA |
| ***Astroloba_rubriflora*** | KJ557835.1_ | NA | GI371438051 | NA | KF013533.1 | GI388268791 | KJ557616 |
| ***Bulbine_semibarbata*** | NA | NA | GI313756479 | NA | AJ290293.1 | NA | NA |
| ***Bulbine_succulenta*** | NA | NA | GI1497828 | NA | AJ290294.1 | NA | NA |
| ***Bulbine_wiesei*** | NA | NA | GI4038482 | NA | AJ290295.1 | NA | NA |
| ***Gasteria_acinacifolia*** | GI37361750 | GI371436915 | GI37361632 | NA | JX630317.1 | GI388268765 | KJ557617 |
| ***Gasteria_batesiana*** | GI33635898 | GI371436916 | GI33635974 | NA | KF013530.1 | NA | NA |
| ***Gasteria_bicolor*** | GI37361748 | NA | GI37361630 | NA | KF013531.1 | GI37361682 | NA |
| ***Gasteria_brachyphylla*** | NA | GI345285493 | NA | NA | HQ646841.1 | GI345285540 | NA |
| ***Gasteria_brevifolia*** | NA | NA | GI555630971 | NA | KF013529.1 | NA | NA |
| ***Gasteria_carinata*** | NA | GI388268505 | NA | NA | JX630309.1 | GI388268769 | KJ557619 |
| ***Gasteria_croucheri*** | GI371437324 | GI371436918 | GI371438069 | NA | JX630318.1 | GI388268771 | NA |
| ***Gasteria_disticha*** | GI371437328 | GI371436919 | GI371438073 | NA | JX630307.1 | GI388268772 | NA |
| ***Gasteria_doreeniae*** | GI371437330 | GI371436920 | GI371438075 | NA | JX630320.1 | GI388268773 | NA |
| ***Gasteria_ellaphieae*** | GI371437332 | GI371436921 | GI371438077 | NA | JX630311.1 | GI388268774 | NA |
| ***Gasteria_excelsa*** | GI33635904 | GI371436922 | GI33635984 | NA | JX630316.1 | GI388268775 | NA |
| ***Gasteria_glauca*** | GI371437338 | GI371436923 | GI371438083 | NA | NA | GI388268776 | KJ557620 |
| ***Gasteria_glomerata*** | GI33635896 | GI371436924 | GI33635966 | NA | JX630304.1 | GI388268777 | NA |
| ***Gasteria_maculata*** | GI33635902 | NA | GI33635968 | NA | NA | NA | NA |
| ***Gasteria_nitida*** | GI371437342 | GI371436925 | GI371438087 | NA | JX630319.1 | GI388268766 | NA |
| ***Gasteria_pillansii*** | GI371437348 | NA | GI371438093 | NA | JX630308.1 | GI388268779 | NA |
| ***Gasteria_polita*** | GI371437350 | GI371436927 | GI371438095 | NA | JX630312.1 | GI388268780 | NA |
| ***Gasteria_pulchra*** | GI371437352 | GI371436928 | GI371438097 | NA | JX630313.1 | GI388268781 | NA |
| ***Gasteria_rawlinsonii*** | GI371437354 | GI371436929 | GI371438099 | NA | JX630306.1 | GI388268782 | KJ557621 |
| ***Gasteria_tukhelensis*** | GI371437356 | GI371436930 | GI371438101 | NA | JX630305.1 | GI388268783 | NA |
| ***Gasteria_vlokii*** | GI371437358 | GI388268507 | GI371438103 | NA | JX630314.1 | GI388268792 | NA |
| ***Gonialoe_variegata*** | JQ024171.1 | NA | GI371438039 | NA | KC985127.1 | NA | KJ557600 |
| ***Haworthia_angustifolia*** | GI371437389 | GI388268509 | GI371438137 | NA | JX630211.1 | GI345285501 | NA |
| ***Haworthia_aristata*** | GI371437415 | GI345285444 | GI371438167 | NA | HQ646791.1 | GI345285502 | NA |
| ***Haworthia_bayeri*** | GI371437429 | GI388268513 | GI371438183 | NA | JX630180.1 | GI388268854 | NA |
| ***Haworthia_blackburniae*** | GI371437433 | GI371436933 | GI371438185 | NA | HQ646793.1 | GI388268720 | NA |
| ***Haworthia_chloracantha*** | GI371437449 | GI388268521 | GI371438199 | NA | JX630291.1 | GI388268857 | NA |
| ***Haworthia_coarctata*** | GI37361756 | NA | GI37361638 | NA | NA | NA | NA |
| ***Haworthia_cooperi*** | GI371437463 | GI345285450 | GI371438215 | NA | KF013521.1 | GI388268721 | NA |
| ***Haworthia_decipiens*** | GI749406093 | GI371436941 | GI371438255 | NA | NA | GI388268727 | KJ557624 |
| ***Haworthia_elizeae*** | GI371437515 | NA | GI371438267 | NA | NA | GI388268729 | NA |
| ***Haworthia_emelyae*** | GI371437517 | GI388268525 | GI371438271 | NA | JX630179.1 | GI388268730 | NA |
| ***Haworthia_floribunda*** | GI371437533 | GI371436946 | GI371438297 | NA | JX630292.1 | GI388268745 | NA |
| ***Haworthia_herbacea*** | GI371437565 | NA | GI371438321 | NA | NA | NA | NA |
| ***Haworthia_icosiphylla*** | GI33635908 | NA | GI33636036 | NA | KF013528.1 | GI37361684 | NA |
| ***Haworthia_magnifica*** | GI371437635 | GI371436952 | GI371438385 | NA | JX630294.1 | GI388268732 | NA |
| ***Haworthia_maraisii*** | GI371437637 | GI345285460 | GI371438387 | NA | HQ646808.1 | GI345285513 | NA |
| ***Haworthia_marumiana*** | GI371437657 | GI371436953 | GI371438407 | NA | JX630212.1 | GI388268742 | NA |
| ***Haworthia_marxii*** | GI371437659 | GI371436954 | GI371438409 | NA | HQ646809.1 | GI388268743 | NA |
| ***Haworthia_mirabilis*** | GI371437703 | GI371436955 | GI371438425 | NA | KF013518.1 | GI388268859 | NA |
| ***Haworthia_monticola*** | GI371437759 | NA | GI371438511 | NA | HQ646810.1 | GI345285515 | NA |
| ***Haworthia_mucronata*** | GI371437765 | GI345285464 | GI371438515 | NA | HQ646811.1 | GI345285516 | NA |
| ***Haworthia_mutica*** | GI371437791 | GI345285465 | GI371438533 | NA | HQ646813.1 | GI388268736 | NA |
| ***Haworthia_outeniquensis*** | GI371437815 | GI371436964 | GI371438567 | NA | NA | GI388268750 | NA |
| ***Haworthia_parksiana*** | GI371437821 | GI345285466 | GI371438571 | NA | HQ646814.1 | GI345285518 | NA |
| ***Haworthia_pubescens*** | GI371437823 | NA | GI371438575 | NA | NA | NA | NA |
| ***Haworthia_pulchella*** | GI371437825 | GI345285468 | GI371438577 | NA | HQ646816.1 | GI345285519 | NA |
| ***Haworthia_pygmaea*** | GI371437833 | GI388268551 | GI371438585 | NA | JX630240.1 | GI388268827 | NA |
| ***Haworthia_reticulata*** | GI371437841 | GI371436967 | GI371438593 | NA | HQ646817.1 | GI388268738 | NA |
| ***Haworthia_retusa*** | GI371437861 | GI371436968 | GI371438607 | NA | JX630220.1 | GI388268739 | NA |
| ***Haworthia_rossouwii*** | NA | GI345285471 | NA | NA | HQ646819.1 | GI345285523 | NA |
| ***Haworthia_semiviva*** | GI371437887 | GI371436969 | GI371438639 | NA | HQ646820.1 | GI388268741 | NA |
| ***Haworthia_sordida*** | GI371437889 | GI388268555 | GI371438643 | NA | HQ646830.1 | GI388268848 | NA |
| ***Haworthia_truncata*** | GI371437895 | GI388268557 | GI371438649 | NA | KF013522.1 | GI388268869 | NA |
| ***Haworthia_turgida*** | GI33635852 | NA | GI33636000 | NA | NA | NA | NA |
| ***Haworthia_variegata*** | GI371437899 | GI388268559 | GI371438655 | NA | NA | GI388268870 | NA |
| ***Haworthia_vittata*** | GI33635848 | NA | GI33635954 | NA | NA | NA | NA |
| ***Haworthia_vlokii*** | GI371437913 | GI371436970 | GI371438667 | NA | JX630215.1 | GI388268752 | NA |
| ***Haworthia_wittebergensis*** | GI371437917 | GI371436971 | GI371438671 | NA | JX630193.1 | GI388268753 | NA |
| ***Haworthia_zantneriana*** | GI371437919 | GI388268563 | GI371438673 | NA | JX630209.1 | GI388268864 | NA |
| ***Haworthiopsis_attenuata*** | GI371437417 | GI388268511 | GI371438169 | NA | HQ646822.1 | GI388268805 | KJ557622 |
| ***Haworthiopsis_bruynsii*** | GI371437445 | GI388268519 | GI371438197 | NA | JX630244.1 | GI388268828 | NA |
| ***Haworthiopsis_coarctata*** | GI386430578 | GI388268523 | GI37361648 | NA | HQ646823.1 | GI388268790 | KJ557623 |
| ***Haworthiopsis_fasciata*** | NA | GI345285476 | GI371438281 | NA | HQ646824.1 | GI388268764 | NA |
| ***Haworthiopsis_limifolia*** | GI371437597 | NA | GI371438349 | NA | JX630185.1 | GI388268835 | NA |
| ***Haworthiopsis_longiana*** | GI371437627 | GI388268539 | GI371438377 | NA | JX630288.1 | GI388268810 | NA |
| ***Haworthiopsis_nigra*** | GI371437799 | GI388268547 | GI371438553 | NA | HQ646828.1 | GI388268846 | NA |
| ***Haworthiopsis_reinwardtii*** | GI371437835 | GI388268553 | GI371438587 | NA | HQ646829.1 | GI388268826 | NA |
| ***Haworthiopsis_venosa*** | GI371437901 | GI388268561 | GI371438657 | NA | HQ646833.1 | NA | NA |
| ***Jodrellia_macrocarpa*** | NA | NA | GI4038595 | NA | AJ290300.1 | NA | NA |
| ***Kumara_plicatilis*** | GI37361728 | NA | GI37361604 | NA | NA | GI388268867 | KJ557627 |
