# Supplementary material for: Diversification rate vs. diversification density: Decoupled consequences of plant height for diversification of Alooideae in time and space
Source: PLoS One. 2020 May 26;15(5):e0233597. doi: 10.1371/journal.pone.0233597 (PMC7250425; doi:10.1371/journal.pone.0233597)
Supplement: S2 Appendix — (DOC) [file pone.0233597.s002.doc]

**APPENDIX S2: MEASURING DIVERSIFICATION DENSITY**

***Rationale and aims***

The average diversification rate in a clade can be measured as *log(Nsp)/t_stem_,* where *Nsp* is the number of species in the clade and *t_stem_* is its stem age (Magallon and Sanderson 2001). This measure reflects the fact that diversification is an exponential process, but it remains a phenomenological measure. It was originally proposed as a good description of phylogenies observed in the fossil record but makes no assumption about the processes of speciation and extinction (Raup et al. 1973).

Our aim here is to find a similar measure for diversification density, the diversity of species in a clade per unit space. While many other processes might influence it, such a measure should be related to the dispersal ability of individuals and to their typical population density in a clade of interest: diversification density should be highest for species with limited dispersal and high population densities since they can undergo speciation over shorter distances due to neutral and selective processes, respectively (see Main text). In addition, we would like this measure to describe the general pattern of diversification per unit space but to be independent of a given speciation mode.

We explored four different practical metrics for quantifying diversification density, in which *Nsp* is the number of species in a clade and *Area* is the total area occupied by the clade:

- *Nsp/Area,* which would be the simplest measure

- *log(Nsp)/Area*, which acknowledges the exponential nature of diversification

- *log(Nsp)/log(Area)*, which rather considers the order of magnitude of the area thanks to the logarithm transformation and is appealing since it relates to how species-area relationships are usually fitted (Arrhenius 1921; Mac Arthur and Wilson 1967)

- *log(Nsp)/sqrt(Area)*, which by using a square-root transformation of area measures the typical linear distance over which diversification occurs (see Discussion for more details)

We used spatially explicit simulations of diversification of a clade to assess the properties of these potential diversification density metrics. We assessed: 1) how well the metric was explained by the simulation parameters (*i.e.,* speciation and migration rates, local population density, total area available for the clade to diversify, mode of speciation), 2) whether the metric possessed some key properties (e.g. that it correlates negatively with migration rate and positively with population density), and 3) whether the metric was independent of the geographic mode of speciation.

***Material and methods***

*Simulation settings*

In order to compare the performance of different possible measures of diversification density we used spatially explicit simulations of the Neutral Theory of Biodiversity, in which individuals reproduce, disperse, and die in a meta-community while they diversify into a clade of multiple species (Hubbell 2001). We used a model that simulates meta-community dynamics in a lattice of pixels (communities) between which the offspring of individuals can disperse (Fig. S2). All technical details of these simulations can be found in the original publication describing this specific simulation model (Boucher et al. 2014), but we summarize its main aspects below:

- The whole landscape in which individuals (and species) evolved was made up of a grid of ***L*** x ***L*** pixels, with ***L*** ranging from 3 to 51 pixels (and always being and odd number so that there is a central pixel in the landscape).
- Local communities had a carrying capacity of ***K*** individuals, with ***K*** ranging from 3 to 500 individuals.
- Each simulation was initiated with an empty landscape except for the central pixel in the domain, which was filled at carrying capacity with individuals of the ancestral species. During the simulation, individuals from this central community reproduced and progressively colonized adjacent empty communities.
- Simulations were run for 1 million steps (each step corresponds to the death of one individual and its replacement by the offspring of another one, see Boucher et al. 2014).
- The per-capita speciation rate ***b*** used in each simulation was a random number uniformly drawn between 1E-4 and 1E-2 events/individual.
- The migration rate ***m*** (*i.e.* the probability that a new individual replacing a dead one comes from a neighboring community and not the focal one) used in each simulation was a random number uniformly drawn between 0 and 1.
- Two speciation modes were used, which occur under different geographic settings (see Boucher et al. 2014 for details). In the vicariance scenario, a linear barrier was randomly drawn in the landscape and divided one focal species into two daughters on each side of this barrier. In the random fission scenario, speciation happened locally and only individuals from one community differentiated into a new species, while the remaining individuals of the ancestral species distributed elsewhere remained another species. We ran 400 simulations in which each speciation event could be of one of these two types, with the parameter ***s*** fixing their relative frequencies uniformly sampled between 0 and 1.

*Analysis of simulation output*

At the end of each simulation, the total number of species present was recorded, as well as the total area of the landscape occupied, measured as the number of pixels in which at least one individual was present.

We then measured how well the different diversification density metrics were explained by different parameters of the simulations: the migration rate ***m***, the per-capita speciation rate ***b***, the dimension of the landscape ***L***, the local carrying capacity of a pixel ***K***, and the proportion of speciation events which occurred under random fission, ***s***. All of these relationships were examined using linear regressions with additive effects of the different explanatory variables (e.g. *Nsp/Area* ~ *m* + *b* + *L* + *K* + *s*).

**Figure S2. Overview of the simulation model.** This scheme describes all the possibilities for a single step of the simulation, in total one million steps were run. Only eight communities with a carrying capacity of three individuals are shown. At the beginning of the step (top of the graph) there are individuals from two different species, represented in red and blue. Some communities are not filled to carrying capacity yet (*i.e.* they have less than three individuals). Arrows show different possibilities, with their associated probabilities given by letters. In case speciation happens, the blue species splits into two: one blue and one purple species. In case speciation does not happen, the parent individual that gives birth to the replacement offspring is circled in black.

***Results***

We first discarded 23 out of 400 simulations in which the total area occupied by the clade at the end of the simulation was 1 pixel, since this would have led to a logarithm of zero.

Tables S1 summarizes results of the simulations and shows how different possible measures of diversification density related to parameters of the simulations.

**Table S1. Measures of diversification density and their relationship with model parameters.** Results for the four possible measures of diversification density are presented in different rows and for each one the following columns give the R-squared of the model and the sign of the coefficients associated with each parameter along with its significance between parentheses (one star p-values < 0.05, denotes three stars p-values < 2.2E-16). Cells in red highlight cases were the behavior of one measure is problematic.

***Discussion***

Spatially explicit simulations of the Neutral Theory of Biodiversity allowed us to investigate how the number of species in a clade and the area it occupies are related under a scenario in which a clade diversifies while colonizing a new area. We are fully aware that these simulations are overly simplistic in that all individuals have the same ecological preferences and dispersal abilities regardless of the species they belong to (Clark 2012). At the same time, the strength of this approach is precisely that it does not make any assumptions regarding the ecology of individuals or the suitability of the environment, thus providing an interesting first-order approximation of reality (Rosindell et al. 2012). Although limited, our simulations also cover different geographic modes of speciation that could be representative of various speciation processes: allopatric speciation driven by geographic isolation (vicariant mode), parapatric speciation driven by divergent adaptation along an environmental gradient (vicariant mode), sympatric ecological speciation (random fission mode) or polyploid speciation (random fission mode).

Results from these simulations allowed us to assess the performance of the four possible measures of diversification density density that we proposed, and to select the one with the most appropriate behavior. Below we detail the characteristics of each possible measure.

*Nsp/Area*

While a large proportion of the variance in *Nsp/Area* is explained by model parameters, measuring diversification density using this index would be problematic because it depends on the frequency of different speciation modes, *s*.

*log(Nsp)/Area*

The second possible measure of diversification density, *log(Nsp)/Area*, correlates in the right direction with *m, b, L* and *K*. However, only a limited proportion of the variance in *log(Nsp)/Area* is explained by model parameters (R-squared = 26.9%).

*log(Nsp)/log(Area)*

A large proportion of the variance in *log(Nsp)/log(Area)* is explained by model parameters but this measure has two undesirable properties for the purpose of measuring diversification density: it depends on the frequency of different speciation modes and is positively related to migration rate.

*log(Nsp)/sqrt(Area)*

Finally and in contrast with other measures, *log(Nsp)/sqrt(Area)* conforms to all our criteria: (i) a large proportion of its variance is explained by model parameters (R-squared=48.5%) ; (ii) it does not depend on the relative frequency of different speciation modes ; (iii) it is negatively associated with both *m* and *L* ; and (iv) it is positively associated with both *b* and *K*.

*Conclusion*

Based on these results, we propose the use of *log(Nsp)/sqrt(Area)* as a measure of diversification density. These simulation results strengthen theoretical arguments. Indeed, the use of *sqrt(Area)* in this formula implicitly acknowledges the diffusive nature of range evolution: under a diffusion process in two dimensions, the extent covered on each dimension (longitude and latitude) grows linearly with the typical diffusion distance and as a consequence the area occupied grows as a quadratic function of the typical diffusion distance. The square root of the *Area* occupied by a clade is in unit of distance and measures the typical diameter of its distribution. Thus, *log(Nsp)/sqrt(Area)* is a measure of the typical linear distance over which diversification takes place.

**References**

Arrhenius, O. 1921. Species and area. J. Ecol. 9:95–99.

Boucher, F. C., W. Thuiller, T. J. Davies, and S. Lavergne. 2014. Neutral biogeography and the evolution of climatic niches. Am. Nat. 183:573–84.

Boucher, F. C., G. A. Verboom, S. Musker, and A. G. Ellis. 2017. Plant size: a key determinant of diversification? New Phytol. 216:24–31.

Clark, J. S. 2012. The coherence problem with the Unified Neutral Theory of Biodiversity. Trends Ecol. Evol. 27:198–202.

Fitzjohn, R. G. 2010. Quantitative traits and diversification. Syst. Biol. 59:619–633.

Fitzjohn, R. G., W. P. Maddison, and S. P. Otto. 2009. Estimating trait-dependent speciation and extinction rates from incompletely resolved phylogenies. Syst. Biol. 58:595–611.

Hartigan, J. A., and M. A. Wong. 1979. Algorithm AS 136: A K-Means Clustering Algorithm. J. R. Stat. Soc. Ser. C (Applied Stat. 28:100–108.

Hubbell, S. P. 2001. The Unified Neutral Theory of Biodiversity and Biogeography. Princeton University Press, Princeton and Oxford.

Mac Arthur, R. H., and E. O. Wilson. 1967. The theory of island biogeography. Princeton University Press, Princeton, New Jersey.

Magallon, S. A., and M. J. Sanderson. 2001. Absolute Diversification Rates in Angiosperm Clades. Evolution (N. Y). 55:1762–1780.

Raup, D. M., S. J. Gould, T. J. M. Schopf, and D. S. Simberloff. 1973. Stochastic Models of Phylogeny and the Evolution of Diversity. J. Geol. 81:525–542. The University of Chicago Press.

Rosindell, J., S. P. Hubbell, F. He, L. J. Harmon, and R. S. Etienne. 2012. The case for ecological neutral theory. Trends Ecol. Evol. 27:203–208.

**Table S2. Models for the evolution of plant height across the Alooideae phylogeny.** The Table shows the AIC weight of all seven models fitted, as well as ML parameter estimates obtained on the MCC tree.
