# Supplementary figures and images for: Diversification rate vs. diversification density: Decoupled consequences of plant height for diversification of Alooideae in time and space
Source: PLoS One. 2020 May 26;15(5):e0233597. doi: 10.1371/journal.pone.0233597 (PMC7250425; doi:10.1371/journal.pone.0233597)

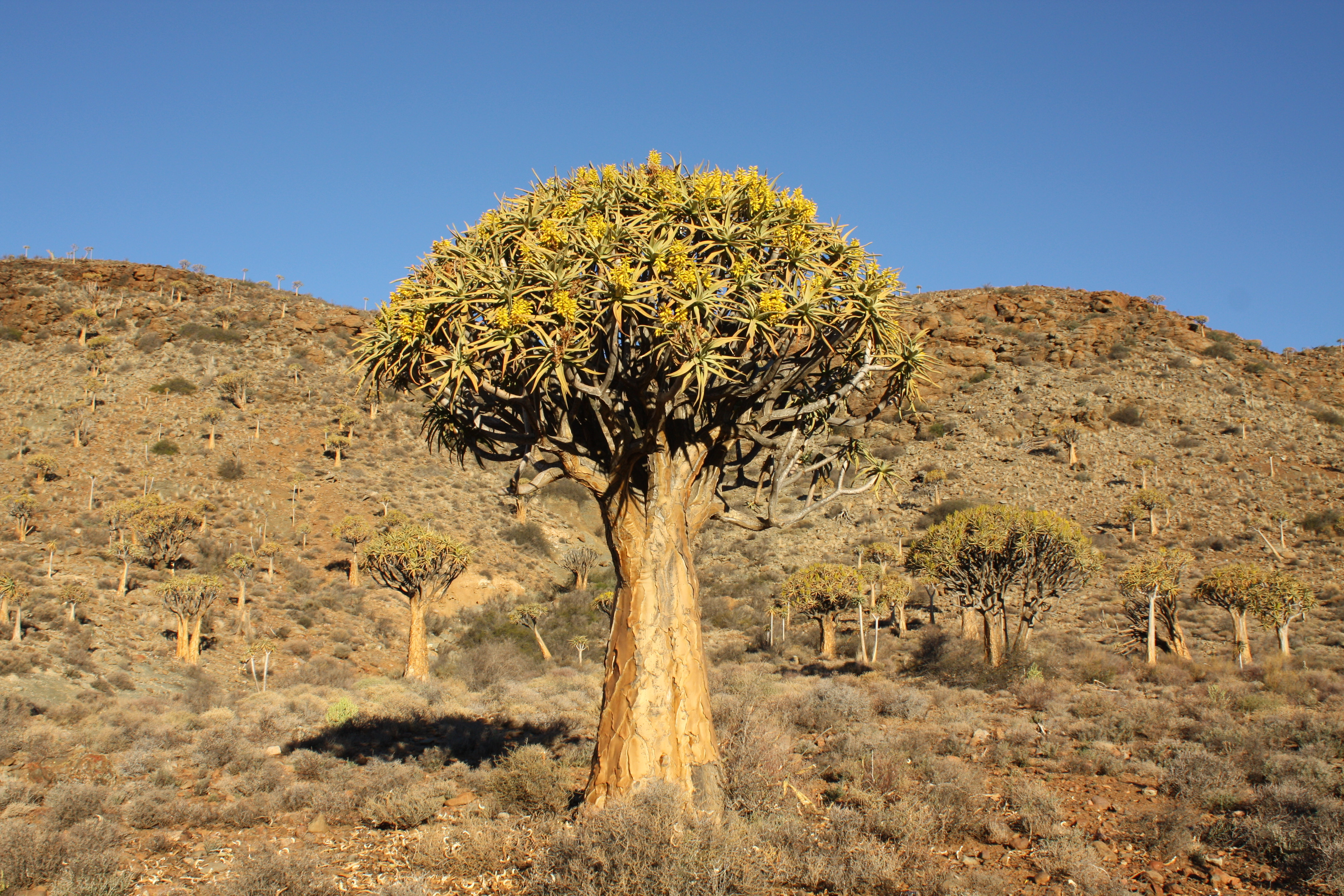

Supplement: S1 Fig — (TIF) [file pone.0233597.s003.tif]
