# Supplementary material for: Diversification rate vs. diversification density: Decoupled consequences of plant height for diversification of Alooideae in time and space
Source: PLoS One. 2020 May 26;15(5):e0233597. doi: 10.1371/journal.pone.0233597 (PMC7250425; doi:10.1371/journal.pone.0233597)
Supplement: Attachment — Submitted filename: Response_Review_Boucher_et_al.docx [file pone.0233597.s004.docx]

Dear Dr. Boucher,

Thank you for submitting your manuscript to PLOS ONE. After careful consideration, we feel that it has merit but does not fully meet PLOS ONE’s publication criteria as it currently stands. Therefore, we invite you to submit a revised version of the manuscript that addresses the points raised during the review process.

Dear authors, I can finally return your paper. I agree with reviewers that you present an intriguing and exciting study on the dynamics of diversification in the Alooideae tribe. Although some points needs to be considered. Looking foward for the next version of your manuscript.

**> We thank the editor for the care paid in reviewing our manuscript. As she will see, we have taken comments by the two reviews seriously and they have helped us improving some key aspects of the manuscript, which lead us to re-run some analyses. We provide a detailed response to each comment below and have submitted a separate document in which all major text changes have been highlighted.**

We would appreciate receiving your revised manuscript by Dec 29 2019 11:59PM. When you are ready to submit your revision, log on to [https://www.editorialmanager.com/pone/](https://www.editorialmanager.com/pone/) and select the 'Submissions Needing Revision' folder to locate your manuscript file.

**> We apologize again for not complying with the initial deadline, but would like to mention that an extension was granted by the handling editor.**

[...]

We look forward to receiving your revised manuscript.

Kind regards,

Juliana Hipólito, Phd
Academic Editor
PLOS ONE

[...]

Additional Editor Comments (if provided):

[Note: HTML markup is below. Please do not edit.]

Reviewers' comments:

Reviewer's Responses to Questions

**Comments to the Author**

1. Is the manuscript technically sound, and do the data support the conclusions?

The manuscript must describe a technically sound piece of scientific research with data that supports the conclusions. Experiments must have been conducted rigorously, with appropriate controls, replication, and sample sizes. The conclusions must be drawn appropriately based on the data presented.

Reviewer #1: Yes

Reviewer #2: Partly

2. Has the statistical analysis been performed appropriately and rigorously?

Reviewer #1: Yes

Reviewer #2: No

3. Have the authors made all data underlying the findings in their manuscript fully available?

The [PLOS Data policy](http://www.plosone.org/static/policies.action#_blank) requires authors to make all data underlying the findings described in their manuscript fully available without restriction, with rare exception (please refer to the Data Availability Statement in the manuscript PDF file). The data should be provided as part of the manuscript or its supporting information, or deposited to a public repository. For example, in addition to summary statistics, the data points behind means, medians and variance measures should be available. If there are restrictions on publicly sharing data—e.g. participant privacy or use of data from a third party—those must be specified.

Reviewer #1: Yes

Reviewer #2: Yes

4. Is the manuscript presented in an intelligible fashion and written in standard English?

PLOS ONE does not copyedit accepted manuscripts, so the language in submitted articles must be clear, correct, and unambiguous. Any typographical or grammatical errors should be corrected at revision, so please note any specific errors here.

Reviewer #1: Yes

Reviewer #2: Yes

5. Review Comments to the Author

Please use the space provided to explain your answers to the questions above. You may also include additional comments for the author, including concerns about dual publication, research ethics, or publication ethics. (Please upload your review as an attachment if it exceeds 20,000 characters)

Reviewer #1: A generally interesting and well done paper that attempts to advance methods in estimating diversification rates by introducing the idea of density. The addition of density is a novel way of interpreting the macroevolutionary scenario and I find it generally to be a useful topic. However, the text neglects to consider the effect of biogeographic history on density. Long distance dispersal in particular can result in situation that decouple density and net diversification rate. For example, when species from the dense area that exhibits high net div disperse to oceanic islands or other low-density areas, a decoupled pattern occurs. This might not be realistic for the study group detailed here, but it is realistic for a lot of other organisms and should be discussed in order to make the topic more broadly relevant.

**> We thank the reviewer for the encouraging comments on our work and on the ideas that we try to introduce in the manuscript. We do agree with the reviewer that historical biogeography is a crucial influence on diversification density, but perhaps this was not made clear enough in the Introduction. The reviewer is probably right that the choice of Alooideae as a case study, with its relatively simple biogeographic history (gradual expansion from Southern Africa across the whole continent plus single colonization of Madagascar and the Arabian peninsula, cf. Grace et al. 2015) does not help to convey the influence of biogeographic history. It is not really clear to us whether in his/her example the reviewer is discussing the density of a clade or of the whole biota of a given region, but we have now rewritten clearly the following sentence of the introduction (edits in uppercase): ‘Diversification density and rate might, however, be decoupled under some conditions THAT DEPEND ON A CLADE’S ECOLOGY AND HISTORICAL BIOGEOGRAPHY’. In addition, we have now written a full paragraph in the Discussion acknowledging the situations in which our proposed metric performs best or worst (L 511-531), which largely discusses the influence of historical biogeography on how to interpret diversification density.**

The elephant in the room, which is not addressed here, and in fact not addressed in many papers attempting to find generally patterns that explain net div is the question of whether the dataset is sufficient to answer the question. The phylogeny used has one clade with high net div, and a couple of clades wth moderately elevated rates. The one clade with a high rate is also the clade with the shortest plants. So, in effect, there are not a lot of opportunities or ‘data points’ that allow for the discovery of a general pattern.

**> We agree with the reviewer that this is an important concern that should always be addressed. His/her judgment on the situation in alooids is partially correct: one clade gives the strongest signal here (the genus *Aloe*), but actually the clade of small plants diversifying fast here is the genus *Haworthia –* in the end, these two points probably give most of the signal. However, rather than using SSE methods which have been shown to be prone to this kind of errors, we did use two the STRAPP randomization test which was proposed by Dan Rabosky and colleagues as a fix to this exact problem. This is probably the reason why we do detect the tendency that we expect from theory (i.e. faster diversification in small plants), but this tendency is not significant. As for the size of the dataset, we had chosen the Alooideae among other Southern African plant groups because this was the largest group for which a decent phylogeny could be estimated (204 spp.), but this might still not be enough to recover the pattern that we expected with statistical support. A sentence summarizing this idea has now been added to the Discussion: ‘In addition, it is possible that the size of the dataset assembled here (204 spp.) is insufficient to recover a significant effect of plant height amidst the many other influences on diversification rate.’**

The scholarship is generally excellent but neglects to cite a number of papers addressing size evolution in plants. Few researchers have attempted to address this topic, so it seems particularly relevant and easy to include these. I have listed a few papers analyzing molecular evolution and diversification rates along with plant size below.

Molecular evolution and plant size:

Smith, S.A. and Donoghue, M.J., 2008. Rates of molecular evolution are linked to life history in flowering plants. science, 322(5898), pp.86-89.

Lanfear, R., Ho, S.Y., Davies, T.J., Moles, A.T., Aarssen, L., Swenson, N.G., Warman, L., Zanne, A.E. and Allen, A.P., 2013. Taller plants have lower rates of molecular evolution. Nature Communications, 4, p.1879.

Diversification rate and plant size:

Sundue, M.A., Testo, W.L. and Ranker, T.A., 2015. Morphological innovation, ecological opportunity, and the radiation of a major vascular epiphyte lineage. Evolution, 69(9), pp.2482-2495.

Ramírez-Barahona, S., Barrera-Redondo, J. and Eguiarte, L.E., 2016. Rates of ecological divergence and body size evolution are correlated with species diversification in scaly tree ferns. Proceedings of the Royal Society B: Biological Sciences, 283(1834), p.20161098.

Testo, W.L. and Sundue, M.A., 2018. Are rates of species diversification and body size evolution coupled in the ferns?. American journal of botany, 105(3), pp.525-535.

> **The first two references on the influence of plant size on rates of molecular evolution are indeed excellent. They are cited in Boucher et al. 2017 New Phytologist, which is the basis for the current paper but we do not really see where these two references would fit in the current paper. We acknowledge that we did not know the literature on ferns that the reviewer refers to. These three papers are indeed very interesting and would have fitted well in our previous review on the processes by which plant size affect diversification rate. However, we do not see a place for citing these papers in the current manuscript whose focus is somewhat different, especially given that we have already >90 references.**

Reviewer #2: The authors present an intriguing and exciting study on the dynamics of diversification in the Alooideae tribe. They formalize the concept of ‘diversification density,’ a spatial counterpart to diversification rate, which may be a useful metric for understanding how lineages assemble into communities or how biodiversity hotspots form. The authors then integrate this new metric with plant height to test the hypotheses that shorter statured species should diversify more rapidly and across smaller spatial distances. They find support for the latter hypothesis only. This manuscript was overall a pleasure to read and I believe it provides an important advancement on our understanding of speciation dynamics across time and space.

Before raising some points that I believe need to be addressed, I wanted to offer a quick apology to the authors for being tardy with my review. It’s taken me a whole month to provide my comments to the editors. The holdup is my fault, not theirs. Sorry!

My primary concerns with this manuscript relate to the methodological. Some of the analyses conducted are very rigorous, cutting edge, and elegant. Others, such as the molecular dating and particularly the treatment of species distributions was cursory and possibly inappropriate.

Regarding the molecular dating, the authors used a single secondary calibration and a deprecated penalized likelihood method. Given the rigor of the ancestral reconstructions and STRAPP analyses, I was surprised that the dating itself was not given more attention. Though the authors suggest that absolute dates are less important in this study because they are concerned with relative diversification rates, this assumes that clade ages are dependent only on the root age. Other, newer PL methods (e.g., chronos, treePL) offer more rigorous data-exploration and cross-validation approaches.

> **The reviewer probably misinterpreted this section since we indeed used a rather new PL dating method : the function *chronos*  as implemented in the *ape* R package. We do agree though that using a single calibration point, and especially a secondary calibration, is far from ideal but as we clearly state in the main text this is the only solution for Alooideae since no fossil has been found for this tribe.**

I have several concerns regarding the species distribution data. First, the authors downloaded species distributions from the SANBI database and apparently proceeded with no further quality control. Species distribution databases – even very good ones – have many errors. Because species ranges are so important to this study, erroneous records are a problem. Second, there is tremendous bias in species collections – both geographically and taxonomically. What steps were taken to ensure evenness of sampling across species and areas? If there is unevenness in collections, then the measure of species area as the sum of QDS is going to be incomparable across species and a different method (convex hull area?) could be more appropriate. Third, the authors restricted their analyses to contiguous Southern Africa. By doing so, they are artificially and unevenly reducing species range sizes (and inflating density) for those species (lineages) that extend beyond contiguous southern Africa.

**> We had indeed chosen to use the SANBI database precisely because of its quality (it is very carefully curated by South African botanists) and for its thorough and even sampling of the whole Southern African region. But we agree with the reviewer’s comments and took his/her critique so seriously that we actually reanalyzed all distributions using another source: occurrence points from the GBIF database, downloaded by the end of December 2019. The advantage of this is that this new dataset covers the whole distribution of Alooideae (i.e. the Arabian peninsula, continental Africa and Madagascar), and thus much more species than the previous Southern African dataset.**

**On the other hand, the known drawback is the poor quality of the GBIF record. To deal with this issue we have produced maps of GBIF records for each species of Alooideae and manually curated them. We were able to clean many records of the following types:**

**- occurrences outside of the native distribution of the subfamily**

**- points from botanical gardens across Africa (especially from Cape Town, Durban and Johannesburg)**

**- points that were outside of the known distribution for the species as defined by Germishuizen & N. L. Meyer (Plants of Southern Africa: An annotated checklist [4th approach to the List of Species of Southern African Plants])**

**We then proceeded with this new dataset and calculated the area occupied by different clades using the convex hull of all known points, as suggested by the reviewer. Results are qualitatively unchanged but are even cleaner than when using QDS data from SANBI: there is a significant negative relationship between the median plant height in a clade and its diversification density, which is consistently recovered over all 100 trees from the phylogenetic posterior and over all time slices used to define clades.**

**We thank the reviewer for this suggestion since we agree that it strengthens our analyses by including major parts of the diversity of Alooideae that were previously omitted (i.e. mainly Eastern African and Madagascan species).**

Minor Comments
I found the framing of this study in the context of biodiversity hotspots to be somewhat confusing. I don’t have a specific request for how it can be approved, but rather simply want to explain why I was confused in case the authors decide some edits would help. Based on the introduction (and abstract) as written, I was expecting analyses about net diversity within areas across lineages (i.e., area-centric analyses), but instead the analyses are about species density within lineages across areas (i.e., lineage-centric analyses). An area-centric analysis would ask, e.g., does the species richness in area of a given size (density) increase as the average species height within that area decreases? Whereas, this study (as I understood it) asks, do shorter statured species diversify more rapidly and across smaller spatial scales than taller species? A number of metrics already exist for the area-centric approach (e.g., those coming out of the Mishler and Laffan labs), so I do appreciate the formalization the authors are providing for the lineage-centric perspective. And, of course diversification density is relevant to the formation of biodiversity hotspots.

**> We appreciate the reviewer’s concern. Indeed, our aim here is to expand the classic area-centric approach to the spatial distribution of biodiversity by explicitly considering the historical dimension of a clade’s spread across space. We have clarified this in the first paragraph of the Introduction in the following sentence: ‘Although diversity patterns may be studied from both an ecological and evolutionary perspective, with the two approaches providing complementary insights [5,6], understanding the origins and origination of diversity is a fundamentally evolutionary challenge that has been most commonly addressed in a clade-centric context.’**

Fourth, the implication of the approach is that the area currently occupied by species is the same area in which the species originated.

**> We do not fully agree here. As stated in the Methods section presenting diversification density and the metric we have chosen to measure it, and as explained in much more details in the Appendix, diversification density is a phenomenological measure: it is simply the ratio of the number of species in a clade by the area it occupies. However, this does not preclude the possibility that some processes like historical range changes have occurred and left their imprint on diversification density. Again, the same applies when measuring diversification rate: extinction is difficult to measure directly, but of course it influences diversification rate Hence to summarize very crudely our view: it is not possible for our metric (and more broadly, for the concept of diversification density) to detect whether of not** ‘**the area currently occupied by species is the same area in which the species originated’, but this is not an assumption either. As already mentioned, the new paragraph that we have added to the Discussion (L. 511-531) should clarify this view while clearly acknowledging the weaknesses of the metric.**

Line 25: consider rephrasing “has influenced” to “is associated with”

**> We agree that in the end our approach is correlative and have rephrased it as suggested.**

Paragraph beginning line 56: This is a fairly cursory literature review.

**> Yes, this was indeed a bit too quick. We have now detailed this paragraph, which now reads:**

**‘Although it has never been formalized as we propose here, the role of space in clade diversification has been widely discussed in the macroevolutionary literature. A clade’s range size, for example, is known to be a key determinant of its diversity [17], with the colonization of a new region often triggering rapid evolutionary radiation thanks to the availability of new ecological opportunity [18]. But smoother expansion of a clade’s range should also lead to a higher species richness thanks to an increase in the environment’s carrying capacity, either in purely spatial terms [19,20] or in terms of environmental heterogeneity [20,21]. In a related corpus of ideas, the theory of island biogeography explains the species-area relationship largely as an outcome of the positive effect of island area on immigration (which frequently leads to anagenetic speciation) and its negative effect on extinction [22,23]. Extensions of this theory even show that *in situ* speciation on islands correlates positively with island area [24]. Finally, global analyses find that the area occupied by a flowering plant family is by far the best predictor of its species richness [25] and a detailed study of the Cyperaceae suggests that high richness in a clade is most readily attained by fine partitioning of species’ ranges [26].’**

Line 65: Even greater than area occupied is the extent to which that area is partitioned by individual species, at least in sedges

**> This is a very good reference that we had missed and which goes deeper in explaining how a clade’s range size influences its richness. Thanks for pointing it to us, we know cite it (see above).**

Line 70: But this is context dependent. Widespread boreal lineages are still species poor.

**> We do see the reviewer’s points and actually the next paragraph gives some (not all) conditions under which diversification density and rate might be decoupled. However, we do not really understand this example: many boreal lineages indeed have low diversification density, but they are not known for having high diversification rates and are thus no clear exceptions to the general expectation presented here.**

Line 84: Not necessarily. Species with reduced dispersal distances tend to have higher diversification rates (Givnish 2010)

**> Agreed, other scenarios like the one proposed here are possible. This is why the sentence starts with ‘for example’.**

Line 86: Not necessarily, would depend on how clades are defined.
**> Again, this paragraph is not meant to list all possible situations : they are too many to be enumerated. We have modified the final sentence of this paragraph in order to clearly reflect this and is now reads (changes in uppercase): ‘Situations in which […] should also, AMONG MANY OTHER SCENARIOS, lead to a decoupling of [...]’.**

Line 135: Thanks for including this. Scripts were well annotated and easy to follow.

**> You’re welcome.**

Study Group section: please also include information on dispersal syndrome (which appears to be very important based on the plant height papers cited). Does dispersal syndrome vary across the study group?

**> Done, on lines 169-170.**

Diversification Density metric: Perhaps this is outside the scope of the study…but it seems that lineage density would be more immediately relevant than species density. Besides the subjectivity and variability of species definitions, either a measure of Faith’s phylogenetic diversity or mean phylogenetic distance would more naturally include the phylogeny than the time slice approach, and also better accommodate incomplete taxon sampling.

**> As the reviewer rightly guessed this is outside the scope of the present study: the paper is already heavy with both theoretical and empirical results. But this is an excellent suggestion that should definitely be tested in a following study.**

Line 336. If these results were unexpected, and previous studies with different results were credible, perhaps Kumara should be constrained as monophyletic.

**> Yes, this would have been a valid strategy. The reason for this odd placement is most likely that the two accessions we used had a lot of missing data. However, this is a minor point since these two species were recovered as the two earliest-diverging species within Alooideae: the rest of the subfamily remains monophyletic (see Fig. 2). We do agree that if one of these two species would have been much more nested within the remaining Alooideae (*i.e.* within the genus *Aloe*), this would have posed serious issues when working at the clade level. Anyways, our core analyses rely on unnamed clades and incorporate phylogenetic uncertainty ; we thus feel that the non-monophyly of *Kumara* in our inference is not an issue for our purposes.**

Line 482: they also on average have shorter generation times as they sooner to reproduce than species that first require vertical growth.

**> Exactly. This is detailed in one of the key references that we cite, which is from the same group of authors (Boucher et al. 2017 New Phytologist) and this is why we chose not to go into this kind of details here.**
